# Phylogenomic Analyses of *Hepatica* Species and Comparative Analyses Within Tribe Anemoneae (Ranunculaceae)

**DOI:** 10.3389/fpls.2021.638580

**Published:** 2021-06-04

**Authors:** Kyu Tae Park, SeonJoo Park

**Affiliations:** Department of Life Sciences, Yeungnam University, Gyeongsan, South Korea

**Keywords:** chloroplast genome, inversion, pseudogenization, phylogenetic analyses, gene loss, rearrangement

## Abstract

*Hepatica* is a small genus of Ranunculaceae with medicinal and horticultural value. We characterized nine complete chloroplast (cp) genomes of *Hepatica*, which ranged from 159,549 to 161,081 bp in length and had a typical quadripartite structure with a large single-copy region (LSC; 80,270–81,249 bp), a small single-copy region (SSC; 17,029–17,838 bp), and two copies of inverted repeat (IR; 31,008–31,100 bp). The cp genomes of *Hepatica* possess 76 protein-coding genes (PCGs), 29 tRNAs, and four rRNA genes. Comparative analyses revealed a conserved ca. 5-kb IR expansion in *Hepatica* and other Anemoneae; moreover, multiple inversion events occurred in *Hepatica* and its relatives. Analyses of selection pressure (*dN*/*dS*) showed that most of the PCGs are highly conserved except for *rpl20* and *rpl22* in *Hepatica falconeri*, *Hepatica americana*, and *Hepatica acutiloba.* Two genes (*rps16* and *infA*) were identified as pseudogenes in *Hepatica*. In contrast, *rpl32* gene was completely lost. The phylogenetic analyses based on 76 PCGs resolved the phylogeny of *Hepatica* and its related genera. Non-monophyly of *Anemone s.l.* indicates that *Hepatica* should be reclassified as an independent genus. In addition, *Hepatica nobilis* var. *japonica* is not closely related to *H. nobilis.*

## Introduction

Chloroplast (cp) is associated with photosynthesis and is considered to have originated as endosymbiotic cyanobacteria (Timmis et al., 2004). The cp is usually uniparentally inherited, with multiple copies per cell, and has a slower evolutionary rate than the nuclear and mitochondrial genomes (Drouin et al., 2008). The cp genome is a circular molecule with a quadripartite structure with two inverted repeats (IR) separated by large single-copy (LSC) and small single-copy (SSC) regions (Palmer, 1985; Jansen and Ruhlman, 2012). The land plant cp genomes are highly conserved in terms of gene content, order, and organization (Palmer, 1991; Jansen and Ruhlman, 2012).

Previous phylogenetic analyses have supported three subtribes in Anemoneae (Ehrendorfer and Samuel, 2001; Mikeda et al., 2006; Wang et al., 2009; Xie et al., 2011; Hoot et al., 2012; Lehtonen et al., 2016; Jiang et al., 2017; Liu et al., 2018b). In Clematidinae, almost all satellite genera of *Clematis* (e.g., *Naravelia* and *Archiclematis*) were nested within *Clematis* in a previous study (Wang et al., 2009; Lehtonen et al., 2016; Jiang et al., 2017; Liu et al., 2018a). In subtribe Anemoninae, there is a discrepancy regarding the classification of *Anemone.* Hoot and Palmer (1994), Hoot et al. (2012), and Hoot (1995) suggested a broad concept for the genus and merged *Hepatica*, *Pulsatilla*, *Oreithales*, *Knowltonia*, and *Barneoudia* into *Anemone* based on their molecular phylogenetic results inferred from nrITS and cpDNA data. Ehrendorfer (1995) preferred a narrow concept and suggested the subdivision of the genus into several genera. Furthermore, Jiang et al. (2017) suggested that *Hepatica* is regarded as an independent genus and *Anemone* sections *Anemonidium*, *Keiska*, and *Omalocarpus* should be subsumed into *Hepatica.* Liu et al. (2018b) suggested that subtribe Anemoninae be separated into at least three genera: *Anemoclema*, *Anemone s.l.* (including *Pulsatilla* and *Pulsatilloides*), and *Hepatica* (including *sect. Omalocarpus, sect. Anemonidium*, and *sect. Keiskea*).

Structural rearrangements and inversions within the cp genome of tribe Anemoneae have been reported based on genetic mapping by restriction enzyme sites (Hoot and Palmer, 1994; Johansson, 1999). Recently, several cp genomes of Ranunculaceae have been published (Park et al., 2015, 2020; Szczecińska and Sawicki, 2015; Li et al., 2016; Park and Park, 2016, 2020; Jiang et al., 2017; Liu et al., 2018a, b; Zhang et al., 2015). Liu et al. (2018a, b) and Zhai et al. (2019) reported the complete cp genome sequences of several members of tribe Anemoneae: *Anemoclema*, *Clematis*, *Hepatica*, *Naravelia*, and *Pulsatilla*; they discovered a 4.4-kb expansion of the IR and multiple inversions across Anemoneae.

*Hepatica* Mill. is a small genus of Ranunculaceae comprising plants that are valuable to medicine and horticulture. *Hepatica* is distinguished from *Anemone* L. by the length of the peduncle and simple and entire leaves (Hoot et al., 2012). *Hepatica* comprises 11 taxa and is distributed in temperate regions of the northern hemisphere (Ulbrich, 1906; Nakai, 1937a, b; Meusel et al., 1965; Tamura, 1995). Although *Hepatica* is widely distributed, most species are local endemics (Jalas and Suominen, 1976). The genus is most diverse in East Asia, with four species and two varieties (Pfosser et al., 2011). Nakai (1937a), Nakai (1952) reported three taxa, including two Korean endemics—Hepatica insularis Nakai and *Hepatica maxima* (Nakai) Nakai—divided into two groups: *Hepatica asiatica* Nakai and *H. insularis* with annual leaves and *H. maxima* with biennial leaves. *H. asiatica* is widespread from the Korean peninsula to Manchuria, *H. insularis* is restricted to southern Korea and Jeju Island, and *H. maxima* is endemic to Ulleung Island. Two taxa—*H. nobilis* var. *japonica* Nakai and *H. nobilis* var. *pubescens* (Hiroe) Hiroe—are considered varieties of *Hepatica nobilis* Mill. and are distributed in Japan (Nakai, 1937a, b; Hiroe, 1957). *Hepatica henryi* (Oliv.) Steward is restricted to central west China (Oliver, 1887; Wang, 1980). *Hepatica falconeri* (Thomson) Yuz. is found in the Kashmir and Pamir regions (Shishkin, 1937; Tamura, 1995; Ogisu et al., 2002). *Hepatica americana* (DC.) Ker Gawl. and *Hepatica acutiloba* DC. occur in central to northeastern North America (Steyermark and Steyermark, 1960). Two *Hepatica* are distributed in Europe: *H. nobilis* var. *nobilis—*the type species of *Hepatica*—is widespread in Europe, and *Hepatica transsilvanica* Fuss is a local endemic in the alpine regions of Transylvania, Romania.

Previous phylogenetic investigations of *Hepatica* have used morphological, cytological, and molecular approaches (Kurita, 1955; Hoot and Palmer, 1994; Ogisu et al., 2002; Woo et al., 2002; Jiang et al., 2018; Zhai et al., 2019). However, only a few species of *Hepatica* have been included (Hoot and Palmer, 1994; Meyer et al., 2010), and relationships within *Hepatica* remain ambiguous (Pfosser et al., 2011; Jiang et al., 2018).

The cp genome has been reported for two *Hepatica* species, *H. henryi* and *H. maxima*. The *Hepatica* cp genome has undergone several inversions, and intracellular gene transfer events were detected (Liu et al., 2018b; Zhai et al., 2019; Park and Park, 2020). Therefore, it is necessary to uncover the cp genome characteristics of *Hepatica* to resolve their phylogenetic relationships. For this purpose, we sequenced, assembled, and analyzed the cp genomes of nine taxa of *Hepatica.* This study aims to (1) identify the genomic characteristics of these taxa, (2) discover their cp genome structures and determine structural variation by comparing them with the cp genomes of nine Anemoneae and one outgroup [*Oxygraphis glacialis* (Fischer ex de Candolle) Bunge], and (3) clarify the phylogenetic relationship of *Hepatica* using 76 protein-coding genes (PCGs).

## Materials and Methods

### Plant Sampling, DNA Isolation, and Sequencing

Nine *Hepatica* taxa were collected from the field, herbaria, or flower companies (Supplementary Table 1). The living material was replanted in the greenhouse of the Yeungnam University Herbarium (YNUH), Gyeongsan, South Korea. We generated chloroplast genome sequences by isolating total genomic DNA from fresh tissue with a DNeasy Plant Mini Kit (Qiagen Inc., Valencia, CA, United States). From the herbarium materials, DNA was extracted using a modified CTAB method (Allen et al., 2006). The sequencing was outsourced to Phyzen^1^ (Seongnam, South Korea), generating 150-bp paired-end reads from a library of 350- and 550-bp inserts on an Illumina Hiseq 2500 platform (Illumina, San Diego, CA, United States).

### Chloroplast Genome Assembly and Gene Annotation

The obtained raw data were filtered using an NGS QC Tool Kit (Patel and Jain, 2012) by trimming the adaptors and filtering low-quality reads using default options. After filtering the raw data, clean reads were assembled using SOAPdenovo2 (Lou et al., 2012). The complete chloroplast genome sequences were annotated using GeSeq with chloroplast genomes of nine Anemoneae species (Supplementary Table 1; Tillich et al., 2017). tRNA genes were verified with the tRNAscan--SE search server^2^ (Lowe and Chan, 2016). PCGs were defined as putatively functional if they followed two criteria: (1) presence of an open reading frame with the complete conserved domain, verified by the NCBI Conserved Domains Database (CDD^3^), and (2) absence of internal stop codons. The circular maps of *Hepatica* chloroplast genomes were drawn using OGDRAW^4^ (Lohse et al., 2013).

### Comparative Analyses of Chloroplast Genomes

The cp genomes of *Hepatica* were compared to nine Anemoneae cp genomes, with one Ranunculeae cp genome as an outgroup (Supplementary Table 1). In order to evaluate similarity, mVISTA was used to compare the cp genome of *Hepatica* species to the other Anemoneae cp genomes with the LAGAN mode, which produces true multiple alignments regardless of whether they contain inversions or not (Frazer et al., 2004). The IR boundaries were illustrated and compared to those of Ranunculeae species. We aligned cp genome sequences using MAFFT (Katoh and Standley, 2013) and examined the sequence divergence among the *Hepatica* species through a sliding window analysis computing nucleotide variability (*pi*) in DnaSP v.5.0 (Librado and Rozas, 2009). For the sequence divergence analysis, we applied a window size of 600 bp with a 200-bp step size. Genes with similar functions were grouped following a previous study to infer the non-synonymous to synonymous substitution rate ratio (*dN*/*dS*; Chang et al., 2006) using PAML v4.9, with *Anemone flaccida* set as the outgroup. Analyses were performed using genes with the same functions (*atp*, *ndh*, *pet*, *psa*, *psb*, *rpl*, *rpo*, and *rps*) and singular genes (*ccsA*, *clpP*, *cemA*, and *matK*). To identify cp genome rearrangements in *Hepatica*, the complete cp genome alignments for 10 *Hepatica* and the references—nine Anemoneae and one *Oxygraphis—*were performed using progressiveMauve v.2.3.1 (Darling et al., 2004) in Geneious Prime 2019. Inverted repeat B was removed from all cp genomes before the alignments. Locally collinear blocks (LCBs) generated by the Mauve alignment were numbered to estimate genome rearrangements.

### Phylogenetic Analyses

Phylogenetic analysis was performed using all the 76 PCGs in the cp genome. The genes were extracted from cp genomes and aligned using MAFFT (Katoh and Standley, 2013); the alignments were then concatenated in Geneious Prime 2019.2.1. We conducted phylogenetic analyses using RAxML, v. 8.2.4, with 1,000 bootstrap replicates for evaluating the node support. These analyses used the GTR model with GAMMA+I, selected by jModelTest, v. 2.1.9. We also used Bayesian inference (BI) implemented in MrBayes, v.3.2 (Ronquist et al., 2012). To determine the best-fitting substitution model, the Akaike information criterion implemented in jModelTest, v. 2.1.9, was used. The GTR GAMMA+I model was selected. Markov chain Monte Carlo analysis was run for 1,000,000 generations. The first 25% of the trees were discarded as burn-in, and the remaining trees were used to generate a majority-rule consensus tree. The maximum likelihood (ML) and BI analyses were visualized using FigTree, v. 1.4.3^5^.

## Results

### Chloroplast Genome Organization

**FIGURE 1:**
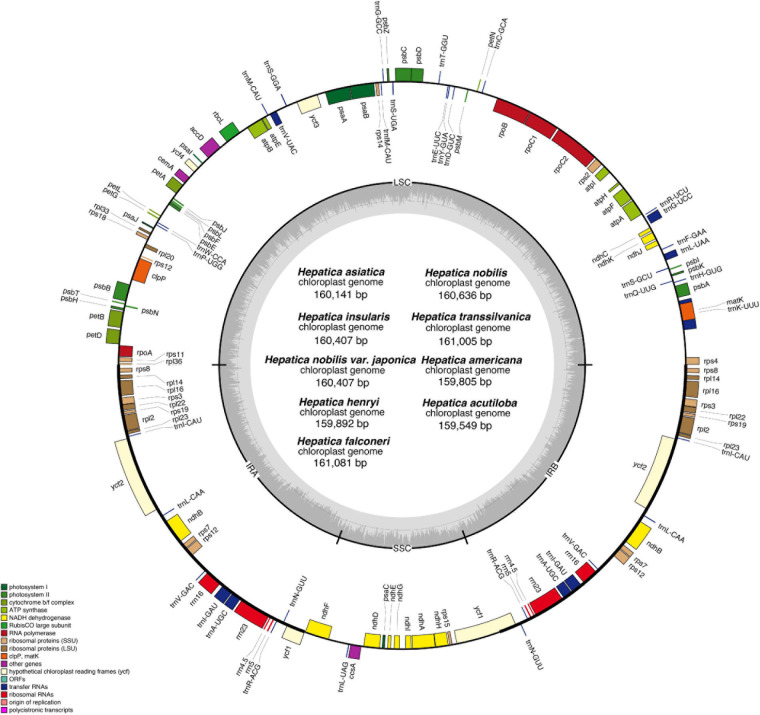
Complete chloroplast genome of genus *Hepatica*. Genes drawn inside the circle are transcribed clockwise, while the genes drawn outside are counterclockwise. The gray plot in the inner circle corresponds to the GC content.

The complete cp genomes of the nine *Hepatica* taxa ranged from 159,549 bp (*H. acutiloba*) to 161,081 bp (*H. falconeri*; Table 1 and Figure 1). The cp genomes had a typical quadripartite structure consisting of LSC 80,270 bp (*H. acutiloba*) to 81,249 bp (*H. falconeri*) in length, SSC 17,029 bp (*H. henryi*) to 17,838 bp (*H. nobilis*) in length, and two copies of IR 31,008 bp (*H. americana*) to 31,100 bp (*H. nobilis* var. *japonica*) in length, respectively (Table 1). The gene content of *Hepatica* cp genome was identical in all species: 76 PCGs, 29 tRNAs, and four rRNAs. Of these 109 genes, 56 were related to self-replication (four in rRNAs and 29 in tRNAs), including eight genes related to large subunits and 11 related to small subunits. Forty-three genes were involved in photosynthesis, including six associated with ATP synthase, 11 with NADH dehydrogenase, six with the cytochrome b/f complex, five with the PSI system, 15 with the PSII system, and one with Rubisco. In addition, nine genes were annotated as having other (*clpP*, *ccsA*, *accD*, *cemA*, and *matK*) or unknown functions (*ycf1*, *ycf2*, *ycf3*, and *ycf4*). Fifteen genes had one intron (*atpF*, *ndhA*, *ndhB*, *petB*, *petd*, *rpl16*, *rpl2*, *rpoC1*, *rps12*, *trnA-UGC*, *trnG-GCC*, *trnI-GAU*, *trnK-UUU*, *trnL-UAA*, and *trnV-UAC*), and two had two introns (*clpP* and *ycf3*; Supplementary Table 2). The GC contents of *Hepatica* cp genomes were 32.2–40.5%.

**TABLE 1:** Characteristics of *Hepatica* cp genomes.

**Species**	**Total**	**Large single-copy**	**Small single-copy**	**Inverted**	**Total GC**	**Protein**	**tRNA**	**rRNA**
	**length (bp)**	**region (bp)**	**region (bp)**	**repeat (bp)**	**contents (%)**	**coding genes**	**genes**	**genes**
*Hepatica asiatica*	160,141	80,343	17,778	31,010	39.2	76	29	4
*Hepatica insularis*	160,470	80,538	17,831	31,019	39.2	76	29	4
*Hepatica maxima*	160,876	80,998	17,684	31,097	39.1	76	29	4
*Hepatica henryi*	159,892	80,779	17,029	31,042	39.2	76	29	4
*Hepatica nobilis* var. *japonica*	160,988	80,996	17,792	31,100	32.2	76	29	4
*Hepatica nobilis* var. *nobilis*	160,636	80,686	17,838	31,056	39.1	76	29	4
*Hepatica transsilvanica*	161,005	81,037	17792	31,088	39.2	76	29	4
*Hepatica americana*	159,805	80,551	17,238	31,008	39.6	76	29	4
*Hepatica acutiloba*	159,549	80,270	17,207	31,036	40.5	76	29	4
*Hepatica falconeri*	161,081	81,249	17,650	31,091	38.9	76	29	4

### Comparative Analyses and Nucleotide Substitution Rates

The mVISTA analysis revealed that the cp genomes of *Hepatica* species were conserved generally across the 10 taxa with a few variable regions, mostly restricted to non-coding regions (Supplementary Figure 1).

The average *pi*-values were estimated to be 0.00262, with a range from 0 to 0.02074 (Supplementary Figure 2). The most variable region was found in the SSC region with an average *pi* = 0.0619. The LSC and IR regions were less variable with *pi* = 0.00323 and 0.00083, respectively. The most variable regions (*pi* > 0.01) included eight intergenic regions (*trnY-trnD*, *trnG-grnS*, *trnR-trnN*, *Ψycf1-ndhF*, *ndhF-trnL*, *trnL-ccsA*, and *rps15-ycf1*) and one coding region (*ycf1*).

**FIGURE 2:**
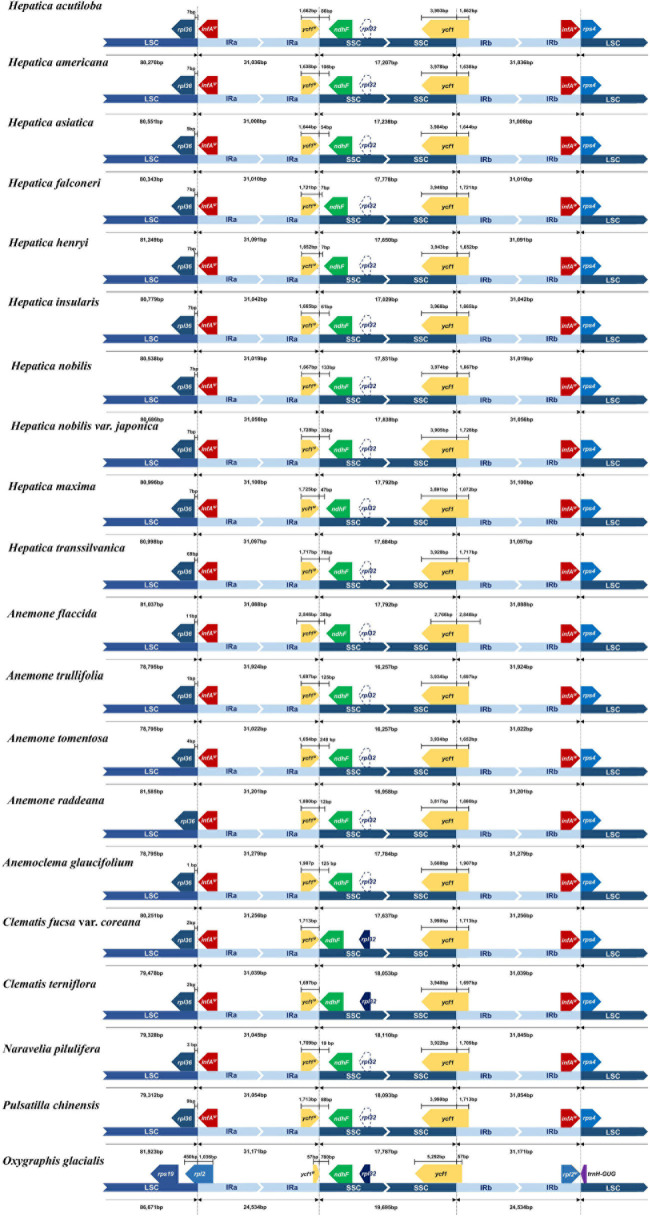
Comparison of the large single-copy region, inverted repeats, and small single-copy region boundaries within tribe Anemoneae.

The length of the IR region ranged from 31,010 to 31,100 bp, and the gene contents of the IR region were conserved in all *Hepatica* species (Figure 2). In *Hepatica*, the LSC/IRa boundary (J_LA_) was located between *rpl36* and *ΨinfA*, and the LSC/IRb boundary (J_LB_) was located on *rps4*. The IRa/SSC and IRb/SSC boundaries (J_SA_ and J_SB_) were located on *ycf1* or between the 5′ ends of truncated *ycf1* and *ndhF.* The IR junction regions of *Hepatica* species are similar to the Anemoneae species. In *Oxygraphis*, the IR junctions (*J*_LA_ and *J*_LB_) were located on *rpl2*, whereas in Anemoneae species, IR regions had been expanded to LSC regions ∼5 kb including *ΨinfA*. Moreover, the IR/SSC boundaries of all Ranunculaceae were located on *ycf1* or between the 5′ ends of truncated *ycf1* and *ndhF*. In this study, the IR expansion event was found to be common to all Anemoneae including *Hepatica*, and the IR expansion has resulted in the duplication of six genes (*rps8*, *rpl14*, *rpl16*, *rps3*, *rpl22*, and *rps19*).

The *dN*/*dS ratios* of most PCGs were less than 1 for all *Hepatica* species and greater than 1 for *rpl20* in *H. acutiloba* (1.6113), *H. americana* (1.6113), and *H. falconeri* (3.5576). The photosynthesis apparatus genes (*pet*, *psa*, and *psb*), ATP synthase gene (*atp*), and RNA polymerase gene (*rpo*) had low *dN*/*dS* ratios (≤0.5), while *atpF* and *petL* in *H. falconeri* had higher *dN*/*dS* ratios (0.7456 and 0.7391, respectively) than in other *Hepatica* species. The RNA processing gene (*matK*) and NADH dehydrogenase gene (*ndh*) showed moderate *dN*/*dS* ratios (≤0.67). *ndhH* and *ndhJ* had low *dN*/*dS* ratios (<0.039). Ribosomal protein genes (*rps* and *rpl*) had a wide range of *dN*/*dS* ratios (0–3.5576). Most of the *rps* and *rpl* genes had moderate *dN*/*dS* ratios, and some genes (*rps7*, *rps8*, *rps11*, *rps12*, *rps19*, *rpl23*, and *rpl36*) had a ratio of 0. The *dN*/*dS* of *rpl22* was 1.1592 in *H. transsilvanica* (Supplementary Figure 3 and Supplementary Table 3).

### Chloroplast Genome Rearrangements and Gene Loss

**FIGURE 3:**
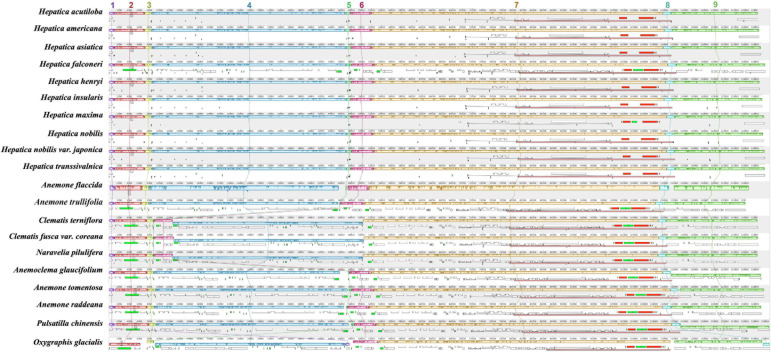
Whole-genome alignment of tribe Anemoneae. Each locally collinear block (LCB) is color-coded and represents a syntenic region. Blocks below the horizontal center line represent inversions relative to the reference (*Oxgraphis glacialis*). The height of the colored region with a block reflects the average sequence identity relative to the reference. The numbers indicate each LCB number.

Nine LCBs identified through whole-genome alignments were shared by all members of tribe Anemoneae and *Oxygraphis* (Figure 3 and Supplementary Table 4). In Anemoneae, gene order is conserved within *Hepatica* and similar to *Anemone*, *Pulsatilla*, and *Anemoclema.* In comparison to *Oxygraphis*, six rearrangement events were detected in Anemoneae: three inversions (LCB_1_, LCB_2_, and LCB_4_) and three relocations (LCB_1_, LCB_5_, and LCB_6_). Among six rearrangements, *Hepatica* shared three inversions with *Anemone*, *Pulsatilla*, and *Anemoclema* (LCB_1_, ∼1.2 kb, including *rps4*; LCB_2_, ∼9.1 kb, including *trnH-GUG*–*rps16*; LCB_4_, ∼49 kb, including *trnG-UCC*–*ycf3*) and two relocations (LCB_1_ and LCB_5_), whereas in *Clematis* including *Naravelia*, additional rearrangements, inversion of LCB_4_, and relocation of LCB_5_ and LCB_6_ (∼4.6 kb, including *trnL-UAA*–*ndhC*) were identified (Figure 3).

We identified two pseudogenes (*infA* and *rps16*) and one gene loss (*rpl32*) in *Hepatica. infA* was a non-functional structure with a 3′ end truncated across the Anemoneae including *Hepatica.* The length of the residual *infA* sequence ranged from 75 to 77 bp (Supplementary Figure 4B). Within Anemoneae, only *Hepatica* was missing a functional *rps16*; exon 1 of the gene was present and conserved in all of the Anemoneae; however, 150 bp of intron and exon 2 were deleted across *Hepatica* species (Supplementary Figure 4A). The *rpl32*, which is located between *ndhF* and *trnL-UAG*, has been completely lost in *Hepatica* and two *Anemone* (*A. flaccida* and *A. trullifolia*), whereas *rpl32* of other Anemoneae was identified as a pseudogene except in *Clematis fusca* var. *coreana* (Supplementary Figures 4C, 5).

### Phylogenetic Analyses

**FIGURE 4:**
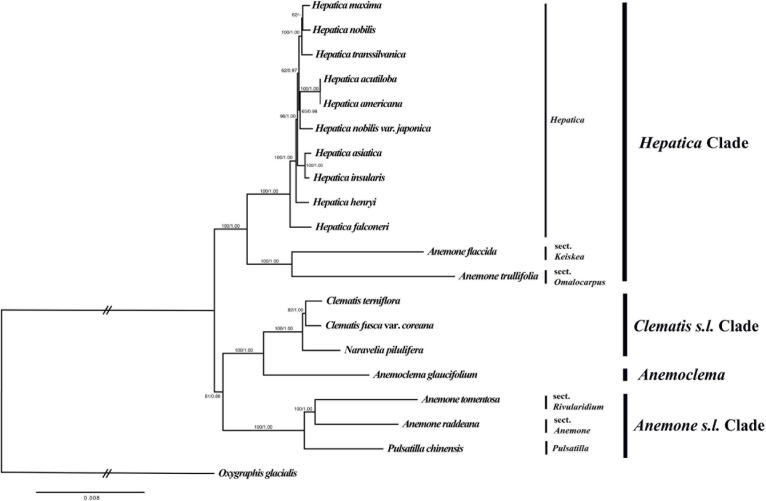
Phylogenetic tree reconstruction of 20 taxa using maximum likelihood based on the concatenated sequence of 76 PCGs. Numbers above the branches indicate bootstrap value and posterior probabilities.

The total alignment length of the nucleotide dataset was 69,400 bp, and the optimal phylogenetic tree in ML analysis had a likelihood score of ln(L) = −151,170.677. The ML tree and Bayesian tree had similar topologies (Figure 4). *Hepatica* formed a monophyletic group and is sister to a clade of *Anemone trullifolia* and *A. flaccida* (BS/PP = 100/1.00). *Anemoclema* was sister to the *Clematis* + *Naravelia* clade (BS/PP = 100/1.00). *Anemone* was not monophyletic. *A. trullifolia* and *A. flaccida* are closely related to *Hepatica*, whereas *A. tomentosa* and *A. raddeana* form a sister clade to *Pulsatilla*; the clade consisting of *Anemone* + *Pulsatilla* is sister to the *Clematis* + *Naravelia* + *Anemonclema* lineage (BS/PP = 61/0.86). Among *Hepatica* species, *H. falconeri* is sister to the rest of the genus. *H. asiatica* and *H. insularis* were grouped as a clade with a high support value (BS/PP = 100/1.00). However, *H. maxima* is sister to *H. nobilis* with weak support (BS/PP = 62/–). *H. nobilis* var. *japonica* was grouped together with *H. acutiloba* and *H. americana* with moderate support (BS/PP = 65/.98). *H. transsilvanica* was sister to the *H. nobilis* and *H. maxima* clade.

## Discussion

### Comparative Characteristics of cp Genome for Hepatica and Its Implication

When compared to other closely related taxa, *Hepatica* has fewer PCGs (76 genes) than other genera (77–78 genes) because of pseudogenization or gene loss of *infA*, *rps16*, and *rpl32* (Zhai et al., 2019). The loss or pseudogenization of three genes (*rps16*, *rpl32*, and *infA*) in the Ranunculaceae cp genome seems to be the result of parallel evolution (Zhai et al., 2019). The *infA* was pseudogenized by truncation, and only 77 bp of the 5′ end of the sequence is remaining in the cp genomes of *Hepatica* and other Anemoneae species. Although pseudogenization of *infA* appeared in several genera of Ranunculaceae, truncation of *infA* was found in only the Anemoneae lineage (Supplementary Figure 4). Usually, *infA* is located in the LSC region in Ranunculaceae, whereas *infA* of Anemoneae is located on the end of IR/LSC boundaries (Figure 2). Thus, it is suggested that IR expansion into the LSC region leads to the truncation of *infA* within Anemoneae lineages. The *rps16* was identified as a pseudogene by deletion of the second exon and intron. The *rps16* pseudogene was also found in only *Hepatica* among the Anemoneae lineage. The existence of the *rps16* pseudogene provides additional molecular evidence that *Hepatica* is monophyletic. Pseudogenization or gene loss of *rps16* has been reported in various lineages, such as *Medicago* (Fabaceae) and *Populus* (Downie and Palmer, 1992; Ueda et al., 2008), and some Ranunculaceae with the loss of complete sequence or frameshift deletion (Zhai et al., 2019; Park et al., 2020), *Draba* (Brassicaceae), and *Lobularia* (Brassicaceae) with deletion of the first exon or deletion of the second exon and intron (Roy et al., 2010), and *Veratrum* (Melanthiaceae) with deletion of the second exon and intron (Do and Kim, 2017). The phylogenetic distribution of the *rpl32* gene loss shows two patterns: (1) a complete loss of all sequences across the *Hepatica* clade and (2) pseudogenization with partial sequences or a frameshift across *Clematis s.l.* + *Anemone s.l.* clade except *Clematis*. Meanwhile, both *rpl32* pseudogenes and intact genes appeared in *Clematis* (Liu et al., 2018a, b; He et al., 2019; Zhai et al., 2019). Therefore, *rpl32* seems to have undergone a gradual gene loss through deletion. The gene loss of *rpl32* has been reported within several lineages of Ranunculaceae (Park et al., 2015; Zhai et al., 2019; Park and Park, 2020). Park et al. (2015) suggested that the reduction of the *ndhF* and *trnL* intergenic spacer (IGS) region is associated with the loss or pseudogenization of *rpl32*. In this study, however, we could not find an affinity between gene loss and length variation of *ndhF* and *trnL* IGS.

Non-functional genes in chloroplast are often associated with functional transfer to the nucleus, such as *rpl32* in Salicaceae and Ranunculaceae (Ueda et al., 2008; Park et al., 2015, 2020; Zhai et al., 2019), *rps16* in *Medicago*, Salicaceae, *Thalictrum*, and Delphinineae (Ueda et al., 2008; Park et al., 2015, 2020), and *infA* in *Arabidopsis*, *Glycine*, *Solanum*, and *Mesembryanthemum* (Millen et al., 2001). However, further investigations that search for transferred genes in nuclear transcriptomes are needed to resolve the fate of missing cp genes.

Structural rearrangements in the chloroplast genomes have been reported in a variety of seed plants, including a 50-kb inversion in Papilionoideae (Doyle et al., 1996), a 22-kb inversion in Asteraceae (Kim et al., 2005), a 42-kb inversion in *Abies* (Tsumura et al., 2000), a 21-kb inversion in Jasmineae (Lee et al., 2007), and multiple inversions in *Passiflora* (Shrestha et al., 2019). We characterized a highly conserved genome structure across Anemoneae including *Hepatica* except for the *Clematis* + *Naravelia* lineage (Figure 3 and Supplementary Figure 1).

Although *Hepatica* cp genomes have an identical structure to those in related taxa, the structural variation compared with *Oxygraphis* could indicate an evolutionary history around the tribal level.

The phylogenetic distribution of arrangements suggests that three inversions (LCB_1_, LCB_2_, and LCB_4_) and two relocations (LCB_1_ and LCB_5_) occurred in the early Anemoneae. On the other hand, the rearrangements in LCB_4_, LCB_5_, and LCB_6_ occurred independently in the *Clematis* + *Naravelia* lineage (Supplementary Figures 5, 6). Repeat analysis identified 30-bp repeats in the flanking regions of LCB_4_ and LCB_5_ in Anemoneae, thus suggesting that these inversions may have been repeat-mediated. Based on these results, the structural rearrangement of *Hepatica* is assumed to have occurred *via* the following four inversions: (1) inversion of LCB_1_ to LCB_5_, (2) inversion of LCB_4_ and LCB, (3) inversion of LCB_2_ to LCB_5_, and (4) the inverted LCB_2_ (Supplementary Figures 5, 6). The *Clematis* + *Naravelia* lineage underwent two additional inversions: inversion of LCB_4_ to LCB_6_ and inversion of LCB_5_ and LCB_6_. The rearrangements in Anemoneae have been reported (Hoot and Palmer, 1994; Liu et al., 2018b; Park and Park, 2020) as we observed four to six inversion events. In addition, the phylogenomic results suggest that the cp genome structure of the ancestor of Anemoneae might be similar to those of *Hepatica*, *Anemone*, *Anemoclema*, and *Pulsatilla* (Supplementary Figures 5, 6).

The synonymous (*dS*) and non-synonymous (*dN*) substitution rate ratios are valuable for understanding molecular evolution (Drouin et al., 2008). A *dN*/*dS* ratio >1, <1, and = 1 indicates positive selection, negative selection, and neutral selection, respectively. Nucleotide substitution rate analyses in the *Hepatica* cp genome revealed that most cp genes are under negative selection (<1). *rpl20* and *rpl22* had significantly high *dN/dS* (>1) in *H. falconeri*, *H. americana*, *H. acutiloba*, and *H. transsilvanica.* The *rpl20* gene in *H. falconeri* had a particularly high *dN*/*dS* ratio (3.5576). Based on this, we presume that natural selection pressure was applied to maintain the protein translation system.

### Phylogenetic Relationships

Ulbrich (1906) suggested that *Hepatica* is divided into two sections based on the crenate lobe: sect. *Hepatica* with an entire lobe (*H. acutiloba*, *H. americana*, *H. asiatica*, *H. falconeri*, *H. insularis*, *H. maxima*, *H. nobilis*, and *H. nobilis.* var. *japonica*) and sect. *Angulosa* with a crenate lobe (*H. henryi*, *H. nobilis* var. *pubescens*, and *H. transsilvanica*). Our phylogenetic tree does not support this classification.

Thomson (1852) described *H. falconeri* as a species of *Anemone*; however, uncertainty remains about its generic position in *Anemone or Hepatica* (Ogisu et al., 2002). Although the leaf shape of *H. falconeri* resembles that of *Anemone*, the morphology of the involucral bracts, pistils, and achenes and the karyotype are closer to *H. nobilis* (Ogisu et al., 2002). According to our study, *H. falconeri* is an early branching species (Figure 4) that features the *rps16* pseudogene, which is only found in the *Hepatica* lineage. Thus, our data support *H. falconeri* as falling into the genus *Hepatica*.

Among Asian *Hepatica*, *H. asiatica* is sister to *H. insularis.* Interestingly, *H. maxima*, a species endemic to Uleung Island, South Korea, is sister to European *Hepatica* (*H. nobilis* and *H. transsilvanica*) rather than Asian *Hepatica*. Previous studies suggested that *H. maxima* originated from populations of *H. asiatica* (Pfosser et al., 2011). However, in contrast with previous results, our phylogenetic analysis shows that *H. maxima* is close to *H. nobilis.*

On the contrary, *H. nobilis* var. *japonica*, an endemic to Japan, is phylogenetically close to the North American *Hepatica. H. nobilis* var. *japonica* was previously classified as *H. acutiloba* before Nakai (1937b), who identified it as a variety of *H. nobilis* based on the shapes of its lobes and bracts. In contrast, Zonneveld (2010) demonstrated that *H. nobilis* var. *japonica* is very similar to *H. asiatica* in genome size and geographically separated from Europe. He also suggested that *H. nobilis* var. *japonica* should be treated as a subspecies of *H. asiatica* (Zonneveld, 2010). Our phylogenetic analysis shows that *H. nobilis* var. *japonica* needs to be elevated to species level rather than treated as a subspecies of *H. asiatica. H. nobilis* var. *japonica* is closer to North American *Hepatica* than it is to *H. nobilis.* However, we could not include *H. nobilis* var. *pubescens*, a Japanese endemic, in this study. To evaluate the classification position of *H. nobilis* var. *japonica*, the relationship between the two Japanese endemics should be investigated further.

In this study, the *Hepatica* is sister to *A. flaccida* (sect. *Keiskea*) and *A. trullifolia* (sect. *Omalocarpus*), whereas *Pulsatilla* is sister to *A. raddeana* (sect. *Anemone*) and *A. tomentosa* (sect. *Rivularidium*). The *Pulsatilla* + *Anemone* clade is close to *Clematis* (including *Naravelia*) and *Anemoclema* (Figure 4). These results are similar to those based on another plastid dataset (Jiang et al., 2017). However, Liu et al. (2018b) found that the *Hepatica* + sect. *Omalocarpus* clade was sister to *Clematis* + *Anemoclema*. Although the topological incongruence was found previously, Anemoneae was divided into three major clades in common. The first clade is subgenus *Anemonidium* of genus *Anemone* including *Anemonidium*, *Omalocarpus*, *Keiskea*, and *Hepatica.* The second clade is the subgenus *Anemone* of genus *Anemone* including *Anemone*, *Barneoudia*, *Knowltonia*, *Oreithales*, *Pulsatilla*, and *Pulsatilloides.* The last clade is *Anemonclema* and *Clematis s.l.*, including *Archiclematis*, *Clematis*, and *Naraverilia.* Based on the nrITS and *atpB-rbcL* dataset, phylogenetic analyses recovered the monophyly of *Anemone s.l.* (Hoot et al., 2012; Jiang et al., 2017), whereas five plastid datasets (*atpB-rbcL*, *matK*, *psbA-trnQ*, *rbcL*, and *rpoB-*trnC) revealed the paraphyly of *Anemone s.l.* (Jiang et al., 2017; Liu et al., 2018b; in this study). According to our study, *Anemone s.l.* is paraphyletic, and our result did not support the classification by Hoot et al. (2012), which placed *Hepatica* into *Anemone.* Thus, the subgenus *Anemonidium* needs to be separated as an independent genus, *Hepatica*, as suggested by Jiang et al. (2017) and Liu et al. (2018b).

## Conclusion

In this study, we sequenced and analyzed the cp genome of nine species of *Hepatica.* The comparative analyses revealed distinct characters of *Hepatica* cp genomes. Even though the cp genome size, genome structure, and gene contents of *Hepatica* were similar to those of other Anemoneae cp genomes, the IR expansion and gene loss or pseudogene demonstrate the evolutionary history of the genus and its relatives. We resolved the monophyly of *Hepatica* and found that some taxa need to be reassessed in tribe Anemoneae. Our results supported that *H. nobilis* var. *japonica* is not closely related to *H. nobilis* and indicated that this taxon needs to be promoted to species level. We identified that *Anemone s.l.* was paraphyletic and recommended that *Anemone s.l.* should be reclassified.

## Data Availability Statement

The datasets presented in this study can be found in online repositories. The names of the repository/repositories and accession number(s) can be found in the article/Supplementary Material.

## Author Contributions

KP and SP conceived and designed the experiments and modified the manuscript. KP performed the experiments, analyzed the data, and prepared a draft of the manuscript and figures. Both authors read and approved the final manuscript.

## Conflict of Interest

The authors declare that the research was conducted in the absence of any commercial or financial relationships that could be construed as a potential conflict of interest.

## Footnotes

1 http://www.phyzen.com

2 http://lowelab.ucsc.edu/tRNAscan-SE/

3 https://www.ncbi.nlm.nih.gov/Structure/cdd/wrpsb.cgi

4 http://ogdraw.mpimp-golm.mpg.de/

5 http://tree.bio.ed.ac.kr/softwar/figtree/
