# Supplementary material for: Phylogenomic Analyses of Hepatica Species and Comparative Analyses Within Tribe Anemoneae (Ranunculaceae)
Source: Front Plant Sci. 2021 Jun 4;12:638580. doi: 10.3389/fpls.2021.638580 (PMC8211876; doi:10.3389/fpls.2021.638580)
Supplement: Supplementary file 2 [file Data_Sheet_1.docx]

Supplementary Material

**Supplementary Table 1.** List of taxa including in analyses

**Supplementary Table 2.** List of gene contents in genus *Hepatica*

**Supplementary Table 3.** Pairwise comparison of nucleotide substitution rates for 76 plastid protein coding genes. *Aneome flaccida* was used as a reference to compare against all *Hepatica* species

**Supplementary Table 4.** Genes in Locally Collinear Block (LCB) identified using ProgressiveMauve alignment for Hepatica cp genomes.

**Supplementary Figure 1. Sequence alignment of 19 *Anemoneae* complete chloroplast genomes.** The chloroplast genome sequences were aligned using mVISTA. The vertical scale indicates the percentage identity, range from 50% to 100%.

**Supplementary Figure 2.** **Plot of sliding window analysis on the complete cp genome for nucleotide diversity (*pi*) compared among ten *Hepatica* species**. The dashed lined are the borders of LSC, SSC and IR regions.

**Supplementary Figure 3. Boxplot showing the variation in pairwise *dN/dS* for *Hepatica* species estimated by comparison with *Anemone flaccida* using PAML.** Each species categorized by functional group. The box presents values between quartiles, the solid lines extended to the minimum and maximum values, and the horizontal lines in the boxes show the median values. (HAC, *H. acutiloba;* HAM, *H. american*; HAS, *H. asiatica*; HFA, *H. falconeri*; HHE, *H. henryi*; HIN, *H. insularis*; HMA, *H. maxima*; HNO, *H. nobilis*; HNJ, *H. nobilis* var. *japonica*; HTR, *H. transsilvanica*)

**Supplementary Figure 4. Alignment of the pseudogenes (*rps16 and infA*) and gene loss(*rpl32*). (**A) Nucleotide alignment of *rps16*-*trnK-UUU* within Anemoneae. (B) Nucleotide alignment of *rps8-rpl36* region. Alignment shows that i*nfA* of *Hepatica* was truncated by 77bp compared with 30 *inf* genes of Ranunculaceae. All Anemoneae *infA* gene are non-functional. (C) Nucleotide sequences alignment of *ndhF-ccsA* region within Anemoneae

**Supplementary Figure 5. Phylogenetic distribution of cp rearrangements events.** The rearrangement events were plotted on the ML tree of *Hepatica* based on 76 PCGs using RAxML.

**Supplementary Figure 6. A putative history of rearrangement across Anemoneae.**


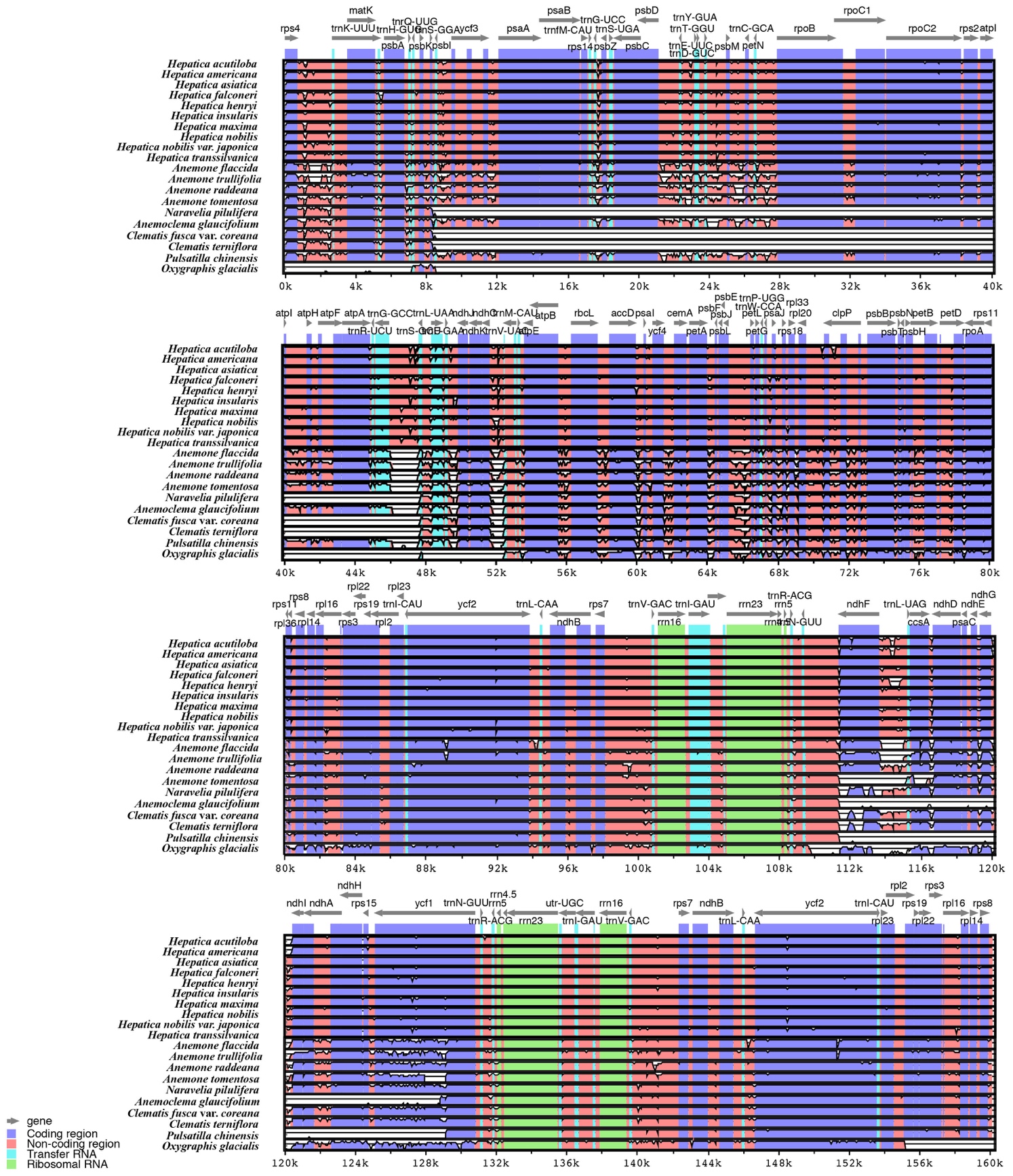


**Supplementary Figure 1. Sequence alignment of 19 *Anemoneae* complete chloroplast genomes.** The chloroplast genome sequences were aligned using mVISTA. The vertical scale indicates the percentage identity, range from 50% to 100%.


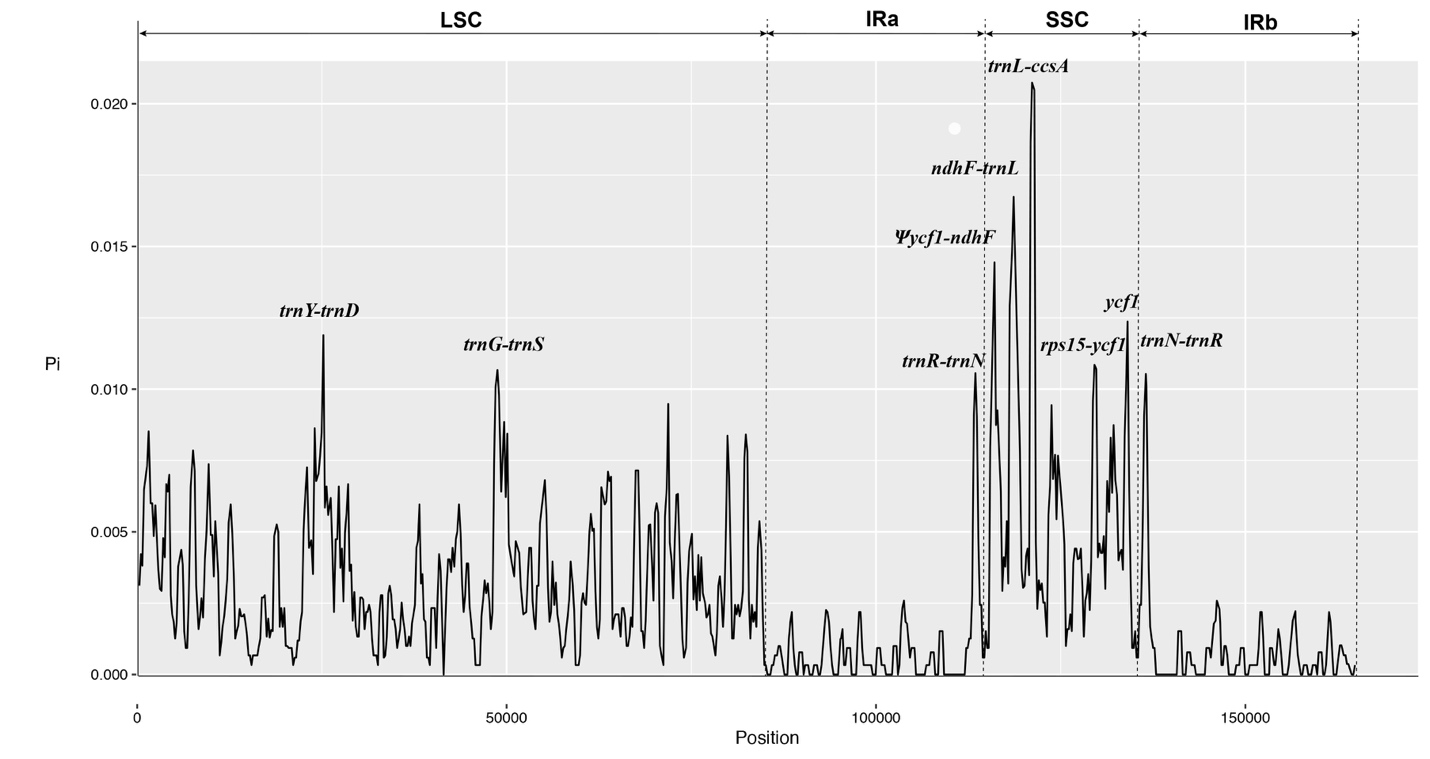


**Supplementary Figure 2.** **Plot of sliding window analysis on the complete cp genome for nucleotide diversity (*pi*) compared among ten *Hepatica* species.** The dashed lined are the borders of LSC, SSC and IR regions.


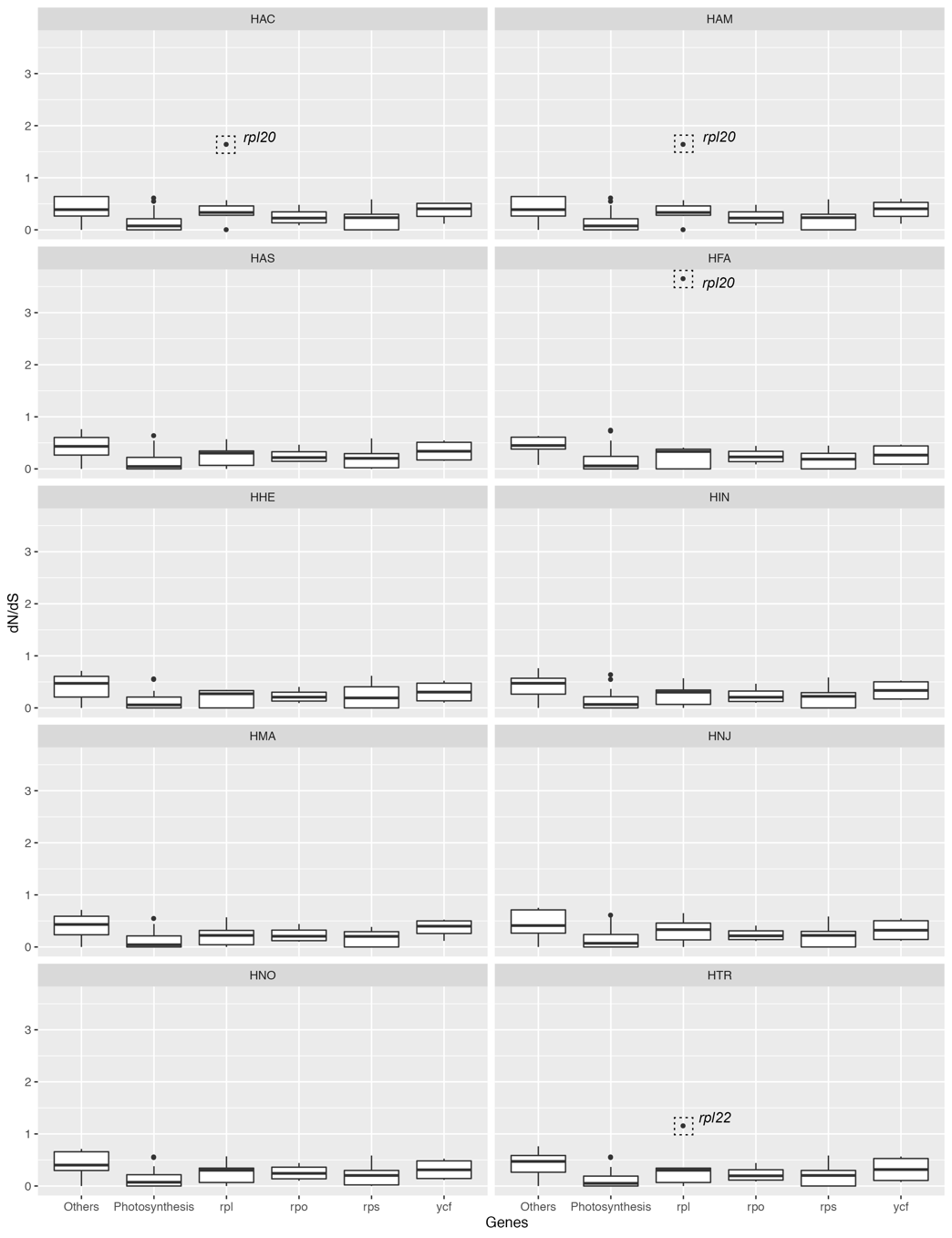


**Supplementary Figure 3. Boxplot showing the variation in pairwise *dN/dS* for *Hepatica* species estimated by comparison with *Anemone flaccida* using PAML.** Each species categorized by functional group. The box presents values between quartiles, the solid lines extended to the minimum and maximum values, and the horizontal lines in the boxes show the median values. (HAC, *H. acutiloba;* HAM, *H. american*; HAS, *H. asiatica*; HFA, *H. falconeri*; HHE, *H. henryi*; HIN, *H. insularis*; HMA, *H. maxima*; HNO, *H. nobilis*; HNJ, *H. nobilis* var. *japonica*; HTR, *H. transsilvanica*)


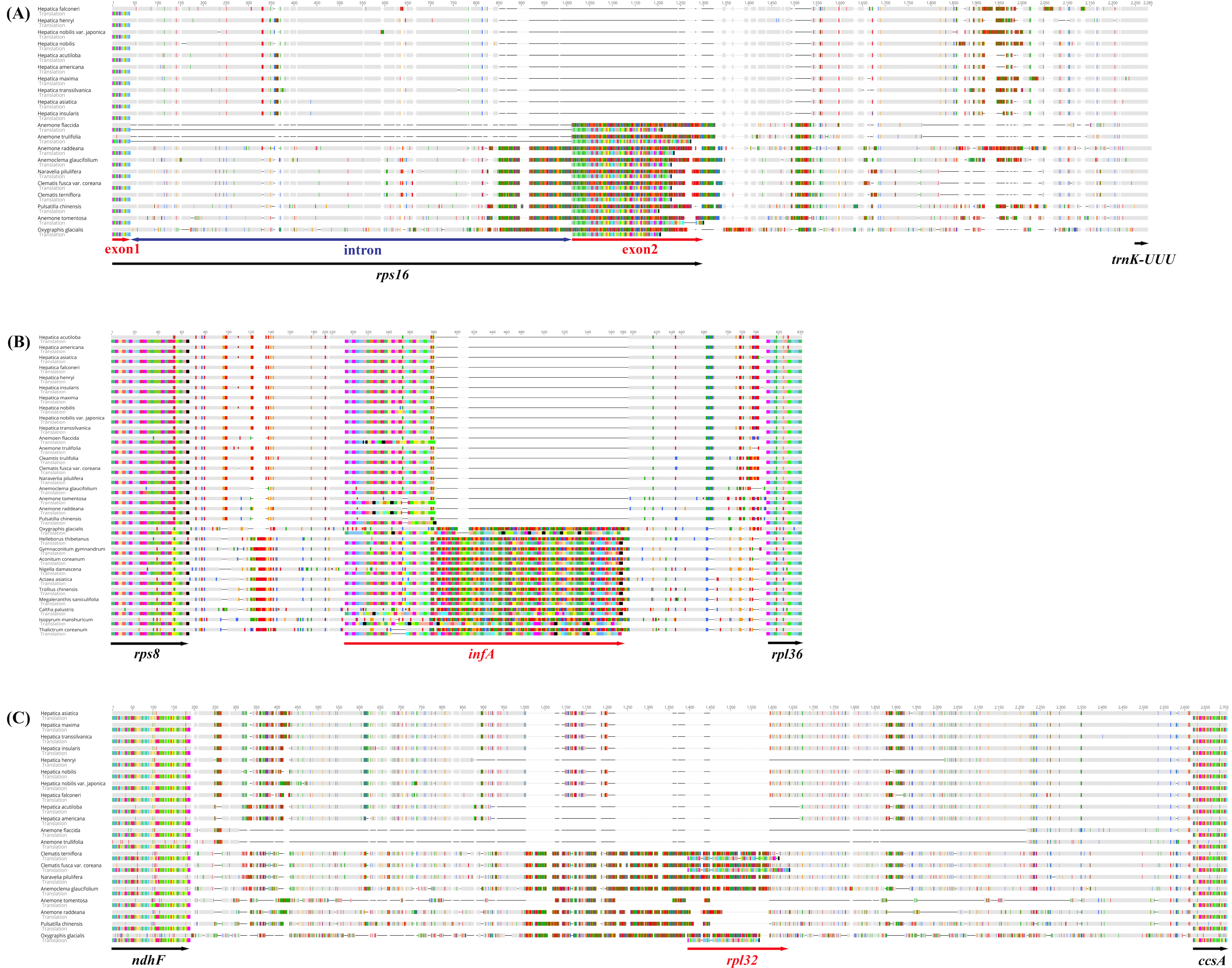


**Supplementary Figure 4. Alignment of the pseudogenes (*rps16 and infA*) and gene loss(*rpl32*). (**A) Nucleotide alignment of *rps16*-*trnK-UUU* within Anemoneae. (B) Nucleotide alignment of *rps8-rpl36* region. Alignment shows that i*nfA* of *Hepatica* was truncated by 77bp compared with 30 *inf* genes of Ranunculaceae. All Anemoneae *infA* gene are non-functional. (C) Nucleotide sequences alignment of *ndhF-ccsA* region within Anemoneae


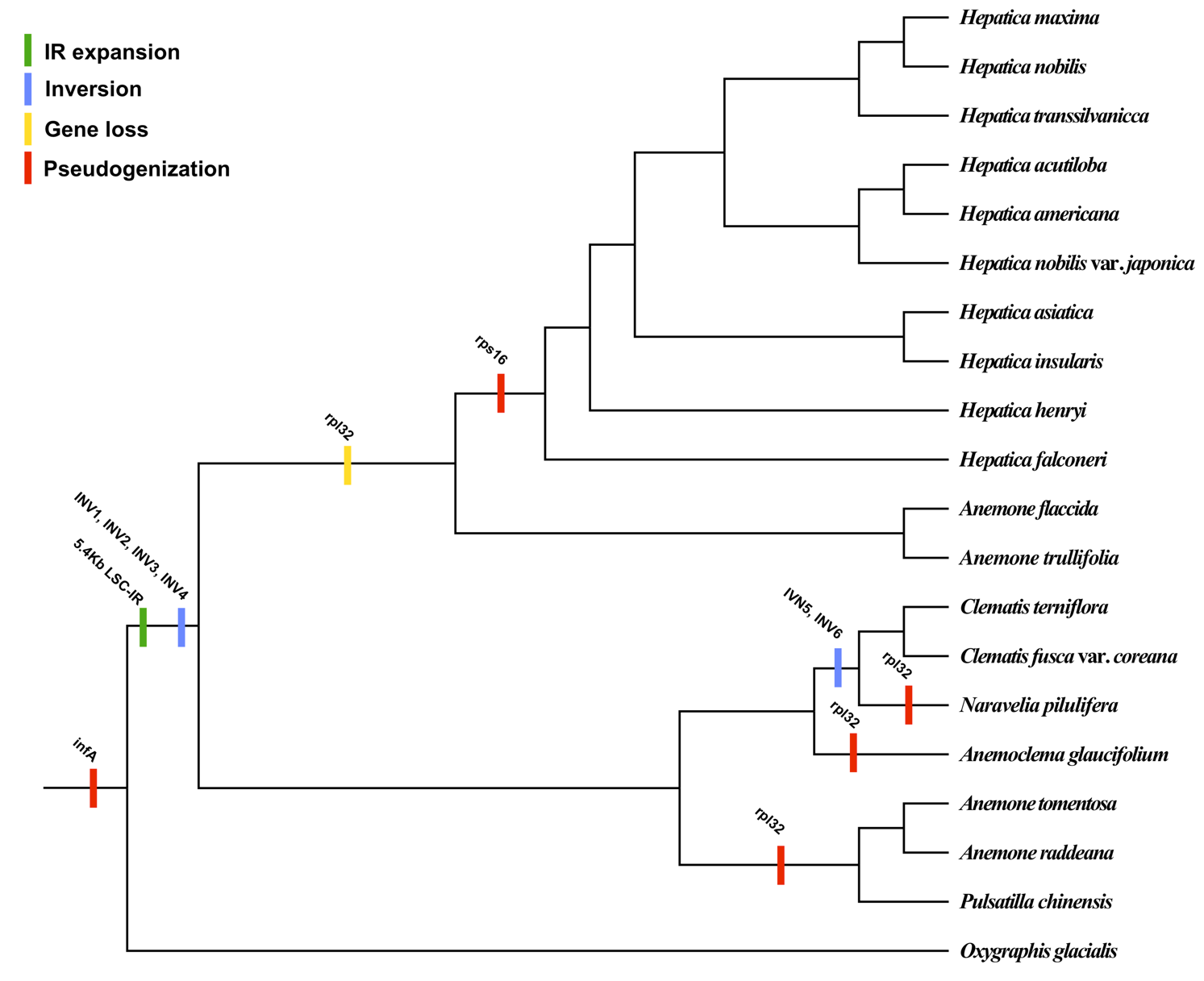


**Supplementary Figure 5. Phylogenetic distribution of cp rearrangements events.** The rearrangement events were plotted on the ML tree of *Hepatica* based on 76 PCGs using RAxML.


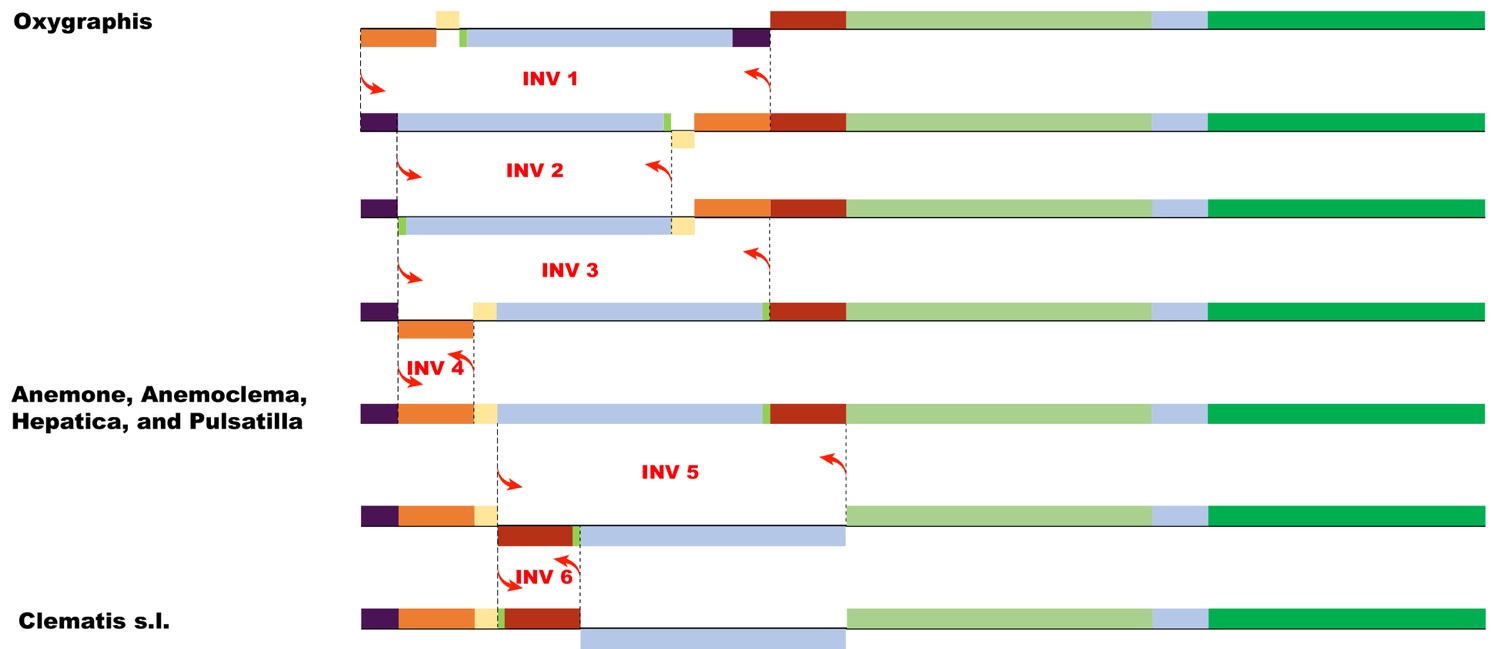


**Supplementary Figure 6. A putative history of rearrangement across Anemoneae.** The inverted regions are indicated by curved arrows. Each box indicated Locally Collinear Block by MAUVE.
